# Tight Junctions in Cell Proliferation

**DOI:** 10.3390/ijms20235972

**Published:** 2019-11-27

**Authors:** Mónica Díaz-Coránguez, Xuwen Liu, David A. Antonetti

**Affiliations:** Department of Ophthalmology and Visual Sciences, University of Michigan, Kellogg Eye Center, Ann Arbor, MI 48105, USA; mdiazcor@med.umich.edu (M.D.-C.); xuwen@med.umich.edu (X.L.)

**Keywords:** tight junctions, cell growth, epithelia, endothelia, proliferation, migration

## Abstract

Tight junction (TJ) proteins form a continuous intercellular network creating a barrier with selective regulation of water, ion, and solutes across endothelial, epithelial, and glial tissues. TJ proteins include the claudin family that confers barrier properties, members of the MARVEL family that contribute to barrier regulation, and JAM molecules, which regulate junction organization and diapedesis. In addition, the membrane-associated proteins such as MAGUK family members, i.e., zonula occludens, form the scaffold linking the transmembrane proteins to both cell signaling molecules and the cytoskeleton. Most studies of TJ have focused on the contribution to cell-cell adhesion and tissue barrier properties. However, recent studies reveal that, similar to adherens junction proteins, TJ proteins contribute to the control of cell proliferation. In this review, we will summarize and discuss the specific role of TJ proteins in the control of epithelial and endothelial cell proliferation. In some cases, the TJ proteins act as a reservoir of critical cell cycle modulators, by binding and regulating their nuclear access, while in other cases, junctional proteins are located at cellular organelles, regulating transcription and proliferation. Collectively, these studies reveal that TJ proteins contribute to the control of cell proliferation and differentiation required for forming and maintaining a tissue barrier.

## 1. TJ Expression in Epithelial Differentiation

**Figure 1:**
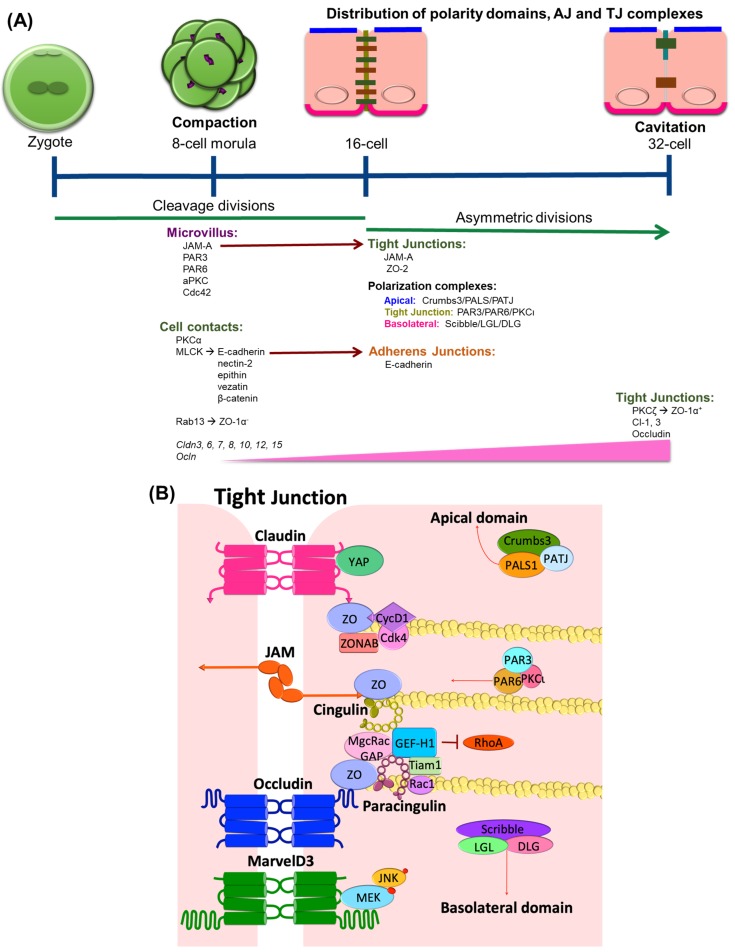
Tight junction proteins in the differentiation of mouse epithelial cells. (**A**) The morula is produced by a series of cleavage divisions of the early embryo, starting with a single cell zygote. Once the embryo has divided into 8 cells, it begins to form a clustered cell mass that expresses AJ and TJ proteins at cell contacts. While JAM and the PAR complex are already expressed at this stage, they localize at the microvillus. As the embryo continues to develop, JAM is recruited into the junctions and polarization complexes are tethered into three specific domains. The subsequent asymmetric divisions initiate the cavitation process at the 32-cell stage. The expression of TJ proteins occludin and claudins increases and together with ZO-1α^+^, they assemble at the junctions, correlating with the differentiation of the first epithelium specialization in the body. (**B**) In fully differentiated tissues, TJ proteins localize at the contact sites of adjacent cells and create a selective paracellular barrier. In addition, the junctional proteins bind and regulate a number of signal transduction factors including those involved in the regulation of cell proliferation. These include sequestering GEF-H1 preventing RhoA activity, the transcription factor ZONAB, and the CycD1/Cdk4 complex, all involved in G1 to S transition. The complexes Crumbs, PAR, and Scribble regulate the proper polarization of apical, TJ, and basolateral domains, respectively.

The role of TJs in growth, proliferation, and differentiation becomes evident in development during epithelial specialization in eukaryotic organisms. Studies of junction formation in early development reveal the contribution of TJs to the early differentiation process and are largely associated with barrier formation. In mice, influenced by the reproductive niche, the germ stem cells undergo the first cell proliferation cycle. The subsequent transcription and translational activity from the embryonic genome during the 2-cell and 4-cell stages is crucial for the first morphogenetic transition in the embryo that occurs during the 8-cell stage, a process that is known as compaction (Figure 1A). Compaction is characterized by the expression and organization of intercellular adhesion and polarization complexes. At a molecular level, protein kinase C isoform alpha (PKCα) and myosin light-chain kinase signaling become activated and maintain cell contact through the regulation of the adherens junction (AJ) proteins E-cadherin, nectin-2, epithin, vezatin, and β-catenin [1,2]. Meanwhile, the membrane-associated guanylate kinase (MAGUK) homolog family member zonula occludens-1 (ZO-1) α^-^, the smaller splice variant of the two ZO-1α isoforms [3], arrives at the blastocyst membrane cell contact where it binds with the Rab-GTPase, Rab13 [4,5,6]. The junctional adhesion molecule (JAM)-A is also expressed at this stage [7] and is localized at the apical microvillous pole together with the partitioning defective (PAR) complex proteins PAR-3, PAR-6, and the atypical PKC isoform iota (aPKCι) as well as Cdc42 [8]. *Cldn3*, *6*, *7*, *8*, *10*, *12*, and *15*, and *Ocln* genes [9] are also expressed in this compaction stage. *Cldn*3 gene shows a particularly high expression compared to other claudins in the progenitors of the trophectoderm during late compaction [10], suggesting a role of claudin-3 protein in differentiation. The TJ protein content increases as the embryo continues to develop and shows an even distribution at the lateral domain similar to the AJs.

The first epithelial specialization differentiation takes places in cavitation, which is a process of the formation of the first cavity in the embryonic body or blastocoel. Cavitation is characterized by asymmetrical divisions and the coordinated polarization of the trophectoderm epithelial cells, a process regulated by junctional components. At the 16-cell stage, E-cadherin assembles at the membrane at cell-cell contact sites and confers the apical and basolateral cell polarization of blastomeres, which leads to a restricted localization of polarity proteins [11]. The polarity complexes Crumbs, which includes Crumbs3, a protein associated with lin seven 1 (PALS1) and a protein associated with tight junctions (PATJ); PAR, including PAR3, PAR6, and aPKCι; and Scribble, including Scribble, lethal giant larvae (LGL), and disc large (DLG), distribute in three specific domains in a mechanism orchestrated by the recruitment of JAM-A into the junctions. JAM-A is recruited to newly formed cell–cell junctions simultaneously with ZO-1. Then, PAR-3 interacts with JAM-A and also tethers to the junction. Although the mechanism is not completely understood, it has been proposed that the JAM-induced localization of the PAR protein complex controls the asymmetrical divisions of the blastocyst [12,13]. Meanwhile, cingulin and ZO-2 assemble to the TJ for the first time [2,14]. At the late 32-cell stage, ZO-1α^+^ [5] is expressed and co-localizes with occludin [15] at the Golgi. Together with claudin-1 and 3, ZO-1α^+^ and occludin assemble at the junctions [16], allowing the embryo to generate a barrier between trophectoderm cells and the nascent blastocoel cavity. The specific function of the ZO-1 α-domain is not completely clear, but a recent study showed that ZO-1α^+^ expression correlates with the grade of Caco-2 cell differentiation. During the exponential phase of Caco-2 cell growth, cells shift in isoform expression from elevated ZO-1α^-^ to elevated ZO-1α^+^ as cells reach confluence [17]. Further, this shift in expression pattern could be manipulated by the cell substrate and correlated with differentiation of the cells, implicating ZO-1α^+^ in differentiation of junctional properties. Little is known about the molecular mechanisms that promote the proper localization of these junctional domains, but PKC isoforms have been implicated through chemical activation of PKC isoform zeta (PKCζ) in trophectoderm cells, which promotes the assembly of ZO-1α^+^ [18], while PKCι interacts with PAR3/6 proteins, promoting localization and positioning at the junctions [19].

The mechanisms of epithelial polarization and differentiation during development differ between species. In addition to mammals, this process has been studied in invertebrate organisms including *C. elegans*, *Xenopus,* and *Drosophila* embryos. More detailed reviews of these species may be found in [11,20]. In contrast with mammals, the polarization of *C. elegans* blastomeres is not directly linked to cell fate specialization since at the 4-cell stage the blastomeres are already polarized but do not form junctions. In fact, the first epithelial specialization of *C. elegans* appears later during organogenesis [21]. In *Xenopus* embryos, both polarization and junction formation start together with the first cleavage, but in this case, the epithelial differentiation process occurs independently of cell adhesion [22]. Distinct from these organisms, the *Drosophila* embryo has a unique cleavage mechanism named cellularization. In this process, the embryo undergoes multiple cell divisions at the same time that are mediated through membrane invaginations. The resultant tightly packed epithelium of 13 columnar hexagonal cells, possesses cytoskeleton-based landmarks that act as localized clusters for AJ and septate junction (SJ) recruitment [23,24]. In *Drosophila*, SJ performs a similar role as TJ. These studies highlight important differences in epithelial junction specializations among species and reveal unique evolutionary resolution of epithelial differentiation.

## 2. TJ Expression in Endothelial Differentiation

In the central nervous system (CNS), the vasculature has unique anatomic and functional properties that contribute to blood-brain (BBB) and blood-retinal (BRB) barriers. The endothelial cells are in close association with neurons, glia, and pericytes that promote the differentiation to a specialized endothelium with TJ proteins that restrict the transport of molecules into the neural tissue. The development of CNS vascularization and angiogenesis begins around embryonic (E) day 9 in rodents. Starting from the perineural vascular plexus, the endothelial network sprouts and covers the entire surface of the neural tube [25].

Endothelial differentiation and specialization in the CNS barriers result from the polarization of the endothelial network and the localization of junctional components along endothelial cell contacts. For the mouse BBB, the differentiation process takes place between E15.5 and E18.5 [26], while the BRB angiogenesis starts after birth, and the barrier properties are established simultaneously with capillary formation between postnatal day 7 and 15 [27]. In humans, both BBB and BRB develop during gestation, but it is not completely clear if the establishment of barrier properties culminate with birth [28]. Although all endothelial cells from the most primitive linage express the TJ protein claudin-5, a junctional protein required for brain barrier properties [29], the sealing of a functional barrier occurs later.

The Wnt/β-catenin signaling pathway is vital for growth and specialization of the CNS vasculature. During mouse brain development, the neuroepithelium-derived Wnt7a/Wnt7b ligands activate signaling through Frizzled-4 (Fzd4) receptor, low-density lipoprotein receptor-related protein 5/6 (Lrp5/6) co-receptor, G-protein-coupled receptor 124 (Gpr124), and reversion-inducing cysteine-rich protein (Reck) co-activators to promote β-catenin activation and angiogenesis [30,31,32]. This signaling is essential for neural tube vascularization because its depletion causes CNS vascular malformation and lethality. Moreover, β-catenin signaling contributes to the specialization of the CNS endothelium by inducing the expression of the glucose transporter and the TJ protein claudin-5 [31]. The non-Wnt ligand norrin activates β-catenin signaling though its receptor Fzd4, the Lrp5/6 co-receptor, and the tetraspanin-12 (Tspan12) co-activator, and this signaling is essential for proper retinal angiogenesis and barrier formation as well as cerebellum barrier formation [33]. In the retina, while Wnt3, Wnt7a, and Wnt7b ligands have only a small contribution in barrier formation [34], the Müller glia-derived norrin is the predominant β-catenin activator that regulates BRB formation and maintenance. Downstream of β-catenin, gene expression of the sex-determining region Y-related high-mobility group box factors 7, 17, and 18 (*Sox7*, *Sox17*, and *Sox18*, respectively) promotes BRB formation [35]. 

## 3. Epithelial-Mesenchymal Transition (EMT)

As part of normal development, epithelial cells transition to mesenchymal phenotype in a process called epithelial mesenchymal transition (EMT). This occurs, for example, during implantation of embryos, as part of gastrulation and neural crest formation. EMT also contributes to both wound repair and to the pathology of neoplasia as a part of tumor dedifferentiation. Under a mesenchymal state, tumor cells are highly invasive and able to migrate to distant sites, establishing metastases. After invading a new tissue, tumors cells may differentiate towards the recovery of their epithelial characteristics in a process called mesenchymal-epithelial transition (MET) [36].

Changes in TJ protein expression and organization are a fundamental step in EMT. Indeed, the most common markers that indicate the epithelial cell transitioning to mesenchymal phenotype include the suppression of TJ protein transcription and loss of junctional complex organization and polarity. These changes coincide with additional changes such as increased synthesis of cytoskeletal components, actin stress fibers, spindle-shaped appearance, increased movement, and resistance to apoptosis [37,38].

**Figure 2:**
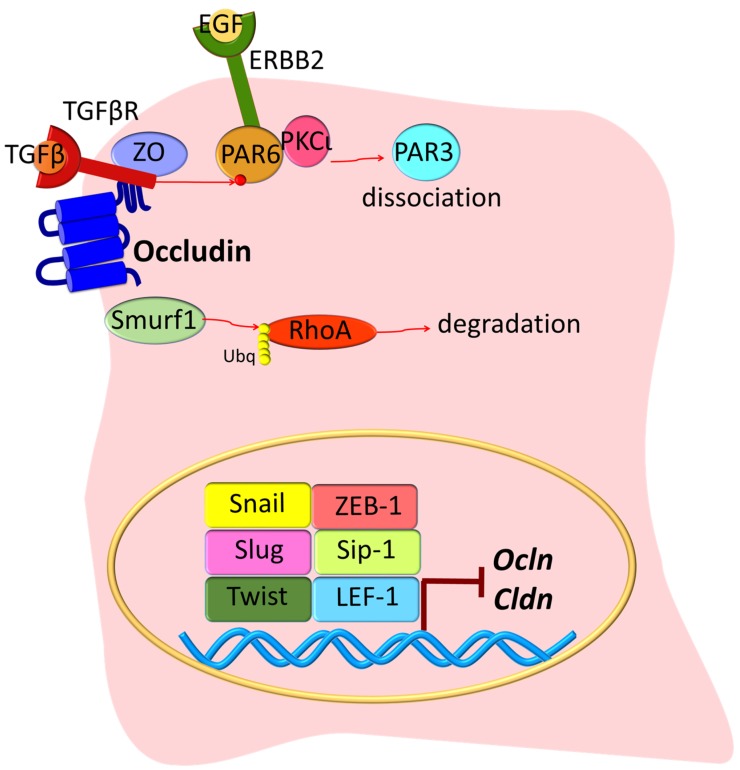
Tight junction proteins in EMT. As an early step in EMT, epithelial cells lose polarity and TJs are disrupted. TGFβ binds its receptor and is recruited to the junction where it interacts with ZO-1 and occludin. TGFβR activation promotes PAR6 phosphorylation. ERBB2 binds to PAR6/PKCι proteins, but PAR3 becomes dissociated from the complex, and this results in overall altered cell polarization. Smurf1 is also recruited into the TJ, where it induces RhoA ubiquitination (Ubq) and degradation. Meanwhile, during EMT, a series of nuclear transcription factors inhibit the expression of TJ genes *Ocln* and *Cldn*. Growth factors including FGF, HGF, and BMPs promote EMT, but the exact mechanism is not completely clear. Together, these molecular mechanisms promote epithelial transformation into a mesenchymal state.

An early step in EMT in cancer cells is loss of gene transcription for junctional components, where the high expression of the transcription regulators Snail, Slug, Twist, zinc finger E-box-binding homeobox 1 (ZEB-1), SMAD interacting protein 1 (Sip-1), and lymphoid enhancer-binding factor 1 (LEF-1) reduces *Ocln* and *Cldn* synthesis [39,40] (Figure 2). With the progression of EMT, the junction complex is disassembled via transforming growth factor beta (TGFβ) signaling. The binding of TGFβ to its receptor TGFβR2 results in its recruitment to the junctional complex where it binds to occludin and promotes phosphorylation of the polarity protein PAR6. Then, the endogenous E3 ubiquitin ligase Smurf1 redistributes to cell junctions and promotes RhoA ubiquitination and degradation, thus leading to cytoskeleton rearrangement and TJ disassembly [41]. Another example is epidermal growth factor (EGF) activation of its receptor (ERBB2), which then interacts with the PAR6-aPKC complex and causes PAR3 dissociation and ultimately TJ breakdown [42]. Other growth factors that promote EMT through their tyrosine kinase receptors include the hepatocyte growth factor (HGF) through its receptor Met; the fibroblast growth factor (FGF); and the bone morphogenetic protein (BMP) [39]. While BMP2 and BMP4 promote EMT [43,44], BMP7 induces MET [45].

While TJ proteins are often reduced in cancers of epithelial origin, in several human cancers, TJ proteins are overexpressed potentially by epigenetic regulation [46]. It is important to note in these cases that the junctional complexes have lost their membrane organization and barrier function. However, their intracellular localization suggests that they might contribute to other cellular functions. Recently, E-cadherin was found to promote metastasis in models of invasive ductal carcinomas [47]. In this work, E-cadherin gene (*Cdh1*) was depleted in MMTV-PyMT invasive ductal carcinoma cells and as expected, this resulted in increased invasion. However, *Cdh1*-null cells also exhibited increased TGFβ/SMAD2/3 and reactive oxygen species signaling, which resulted in reduced cell proliferation, lower survival, and inhibition of metastasis. These studies suggest that E-cadherin acts as a survival factor in invasive ductal carcinomas by limiting reactive oxygen-mediated apoptosis and highlight the non-barrier function of this adherens junction protein. 

## 4. Endothelial-Mesenchymal Transition (EndMT)

Similar to epithelial cells, endothelial tissues have the plasticity to transition to a mesenchymal phenotype (EndMT), and this transition is commonly observed in vasculogenesis. EndMT was first described by Leonard M. Eisenberg in 1995 [48], and recently, considerable attention has been paid to EndMT due to the vital role it plays in human diseases, including cerebral cavernous malformation (CCM) [49] ischemic stroke, atherosclerosis, cardiac fibrosis associated with diabetes mellitus, nephrosis, pulmonary arterial hypertension, diabetic nephropathy, alcoholic liver disease, multiple myeloma, and other cancers [50]. CCM is caused by loss of function mutations in *CCM* genes 1, 2 or 3. The *CCM* gene products bind to the endothelial adherens junction complex in the cytoplasm [51]. In CCM, increased TGFβ and BMP signaling and the consequent EndMT in *CCM1*-null endothelial cells are crucial events in the onset and progression of the disease [49]. Further, recent evidence reveals limited *CCM3*-null endothelial cell clonal expansion induces EndMT in wild-type cells [52]. Importantly, while in epithelial cells all three TGFβ isoforms can induce EMT [53,54], EndMT is primarily stimulated by the TGFβ2 isoform [55,56,57], by BMP2 or by BMP4 [43,44].

In cancer, it has been proposed that EndMT may contribute to metastasis. The cancer microenvironment, which is rich in growth factors secreted by stromal cells, promotes a sustained tip phenotype in most of the endothelial cells undergoing angiogenesis in tumors [58]. Moreover, recent studies suggest that TGFβ induces EndMT in brain endothelial cells [59]. In this study, cells with the mesenchymal phenotype had better adhesion to melanoma cells with increased migration potential, suggesting that EndMT might facilitate brain metastasis.

In some diseases, EndMT remains controversial. Lineage tracing studies employing multiple independent murine Cre lines suggest that fibroblasts do not originate from hematopoietic cells, endothelial cells (through EndMT) or epicardial cells (through EMT) but proliferate from resident fibroblast lineages [60]. Moreover, only few studies have described the presence of EndMT in vascular cells with endothelial barriers. The difficulty in identifying EndMT may be due to the presence of various differentiated states within the growing vessel. Loss of the endothelial markers CD31, von Willebrand factor (vWF), and vascular endothelial cadherin (VE-cadherin) and an increase in the mesenchymal markers alpha smooth muscle actin (α-SMA), fibroblast-specific protein 1 (FSP1), and vimentin have been considered a hallmark of EndMT. However, it has been shown that some level of endothelial markers such as VE-Cadherin, Tie-1, vWF, and cytokeratins is still detected in cells after EndMT. In addition, these markers have heterogeneous expression within the same vessel. In CNS vessels undergoing angiogenesis, the tip cells have a mesenchymal character that allows them to migrate [61,62], suggesting that EndMT may occur at the leading edge of the branching vessels. While tip cells have a fibroblastic phenotype, the intermediate vascular stalk cells proliferate but also have a more differentiated character and contact with neighboring cells. As such, tip cells demonstrate low expression of the TJ protein claudin-5 but high expression of the transcytosis marker PLVAP (plasmalemma vesicle-associated protein). However, the stalk cells simultaneously proliferate and form a barrier through the high expression of claudin-5 [33]. Better knowledge about the mechanisms that control EndMT might help in the development of new therapies in several diseases.

While extensive research has elaborated a role for AJ proteins in the control of epithelial (reviewed in [63]) and endothelial (reviewed in [51]) cell proliferation, growing evidence now implicates the TJ proteins in cell cycle control and regulation of proliferation. 

## 5. Role of Tight Junction Scaffold Proteins in Controlling Cell Proliferation

### 5.1. Zona Occludens (ZO)

ZO proteins serve as links between the transmembrane TJ proteins and the cytoskeleton. ZO proteins bind to the TJ proteins claudins, occludin, and JAMs (Figure 1B) as well as other ZO proteins, promoting the polymerization of the junctional complex and binding cytoskeletal-associated proteins α-catenin and afadin (AF6) linking the TJ to the cytoskeleton [64,65,66]. ZO proteins are part of the MAGUK family, and there are three genes that encode these proteins: ZO-1, -2, and -3 [67,68,69]. Although they have redundant functions, knockout mice studies suggest a role of ZO-1 and -2 in early development. Mice deficient of ZO-1 gene (*Tjp1*) die in E10.5 due to embryonic defects mediated by apoptosis in the neural tube, the notochord, and the allantois areas, as well as extra-embryonic defects in the angiogenesis of the yolk sac, suggesting that ZO-1 regulates tissue organization and remodeling in both epithelial and endothelial tissues [70]. Similarly, mice deficient of ZO-2 gene (*Tjp2*) die shortly after implantation due to an arrest in early gastrulation [71].

**Figure 3:**
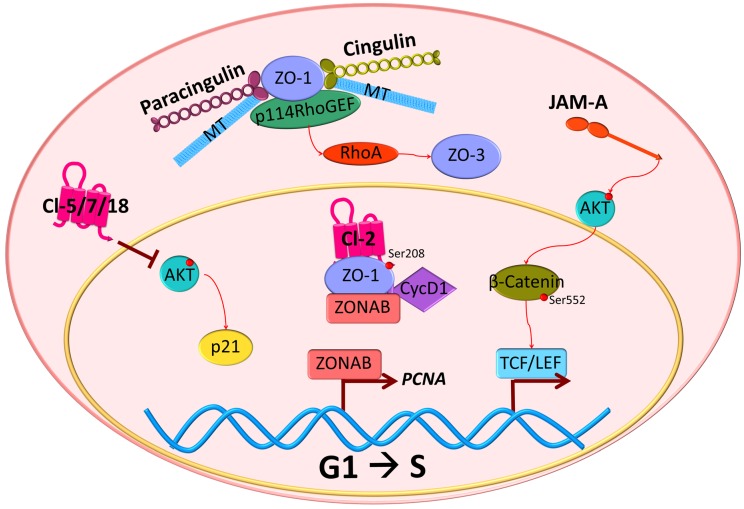
Tight junction proteins in G1/S transition. TJ proteins have been implicated in the control of cell cycle progression. Claudin-5, -7 or -18 (Cl-5/7/18) expression in cancer cells inhibits G1/S transition associated with AKT activation. Alternatively, cell cycle progression can be activated by cingulin and paracingulin dissociation from the junction, which results in a conformational change that allows interaction with microtubules (MT), ZO-1, and p114RhoGEF, promoting RhoA activation and ZO-3 and claudin-2 accumulation that are related to increased G1/S transition. ZO-1 also has been found in nuclear fractions and interacts with claudin-2 (Cl-2) phosphorylated at Ser208, CycD1, and ZONAB. The latter, in sparse cells, can promote *PCNA* gene expression and increase proliferation. In mice deficient of JAM-A gene (*F11r*), AKT becomes activated and phosphorylates β-catenin on Ser552. As a result, β-catenin translocates to the nucleus and activates transcription mediated by TCF/LEF.

Recent publications suggest that ZO proteins are able to transmit information about the degree of cell-cell contacts to the nucleus, thus maintaining a balance between proliferation and differentiation. ZO -1, -2, and -3 sequester key regulators of cell cycle progression at the junction sites [72] (Figure 1B), including ZO-1-associated nucleic acid binding protein (ZONAB). At low-density cell numbers, the Y-box transcription factor ZONAB is located at the nuclear fractions, promoting the expression of G1/S-phase transition through regulation of proliferating cell nuclear antigen (*PCNA*) gene expression (Figure 3). However, at higher density and cell contact, ZO-1 sequesters ZONAB at the TJ, reducing nuclear concentration and thus controlling cell proliferation [73,74]. Cyclin D1 (CycD1) is also sequestrated at the junctions by ZO proteins, together with its associated cyclin-dependent kinase 4 (Cdk4). CycD1 forms a complex with the ZO-3 protein, specifically through its PDZ-binding motif. This interaction regulates epithelial cell proliferation by CycD1 stabilization at the membrane during cell proliferation, since knockdown of ZO-3 gene (*TJP3*) with siRNA results in G0/G1 cell-cycle arrest [75].

**Figure 4:**
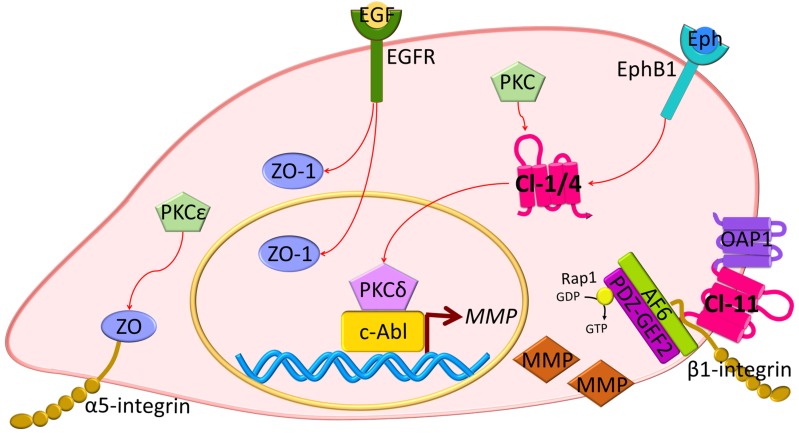
Tight junction proteins in migration and invasion. Overexpression of TJ proteins has been associated with the promotion of cell migration and invasion in cancer cells. EGFR expression has been correlated with ZO-1 localization at nuclear and cytoplasmic fractions, and inhibition of EGFR phosphorylation leads to relocalization of ZO-1 to cell-contacts. PKCε promotes ZO interaction with α5-integrin. Claudin-1 (Cl-1) activates PKCδ, which in turn, binds to c-Abl transcription factors and activates *MMP* transcription. MMPs are secreted and induce basal membrane degradation, increasing the invasive potential of cancer cells. Similarly, EphB1 receptor phosphorylation has been associated with claudin-4 (Cl-4) altered expression promoting MMP expression and secretion. Claudin-11 (Cl-11) interaction with OAP1 and β1-integrin increases cell migration through AF6 and PDZ-GEF2 interaction and Rap1 activation.

Other studies reveal changes in ZO proteins in carcinogenesis. The ZO-1 protein has been found to be highly expressed in adenocarcinoma samples, as compared with healthy tissue [76]. Also, in pancreatic cancer cells, EGFR activation correlates with ZO-1 protein localization at the cytoplasm or at the nucleus, and inhibition of EGFR with AG1478 induces the redistribution of ZO-1 to the junctions [77] (Figure 4). In lung cancer, PKC isoform epsilon (PKCε) activation regulates the interaction between α5-integrin and ZO-1, and this correlates with poor prognosis [78]. Moreover, in EMT, receptor serine/threonine kinase TGFβR type II and I co-localize with ZO-1 at the junctions in a mechanism dependent on TGFβ stimulation and the phosphorylation of PAR6 by TGFβRII [41] (Figure 2). Collectively, these studies suggest that ZO proteins contribute to contact regulated control of cell proliferation.

### 5.2. Cingulin 

Cingulin is a cytoskeletal adaptor protein that has a crucial role in transducing the mechanical force generated by the contraction of the actin-myosin cytoskeleton into functional regulation of the epithelial and endothelial barriers [79]. Its localization at the junctions is mediated by the interaction with the TJ proteins ZO and JAMs, along with its anchoring to the actin cytoskeleton (Figure 1B). 

Recent studies have demonstrated a role of cingulin in cell proliferation and migration through its ability to interact with microtubule (MT)-associated small GTPase activators of RhoA, such as the guanine nucleotide exchange factor H1 (GEF-H1) [80,81,82,83]. Knockdown of cingulin gene (*CGN*) in Mardin-Darby Canine Kidney (MDCK) cells results in increased expression of claudin-2, ZO-3, and RhoA activation (Figure 3). While the knockdown did not affect TJ protein localization or its barrier function, loss of *CGN* increased RhoA-induced G1/S phase transition through its interaction with GEF-H1 [84]. During neural tube closure, the pre-migratory neural crest cells initiate EMT by TJ disruption and cingulin-induced delamination in the neuroepithelium. Both depletion and overexpression of *CGN* increase the migratory neural crest cell population, associated with loss of basal lamina and disruption of the neural tube [85]. Moreover, cingulin participates in cell polarization in epithelial cysts by interacting with the Rab11 family interacting protein 5 (FIP5), an effector of Rab11 GTPase. Cingulin serves as the tethering factor to ensure the fidelity of apical endosome targeting the apical membrane initiation sites [86]. 

### 5.3. Paracingulin

Paracingulin, cingulin-like 1 or the junction-associated-coiled-coil protein (JACOP), is a scaffold TJ protein that maintains the integrity of the association between the MT cytoskeleton and cell junctions [87,88]. With 39% sequence homology to cingulin, paracingulin also interacts with ZO-1 at the globular head domain, promoting its localization at the junctions [89].

Similar to cingulin, paracingulin activates RhoA GTPases to promote cell proliferation. Through its interaction with Rac1, paracingulin promotes the recruitment of Tiam1 (T-cell lymphoma invasion and metastasis 1) and GEF-H1 activators at the junctions (Figure 1B). Therefore, a reduced paracingulin expression in MDCK cells increases RhoA activity and promotes G1/S phase transition [90]. However, when the Rac1 inhibitor MgcRacGAP is present, this interacts with both cingulin and paracingulin at TJs and reduces cell proliferation [91].

Recent studies on endothelial cells suggest a role of paracingulin in angiogenesis. In a primary culture of human dermal microvascular endothelial cells (HDMEC), ZO-1 promoted the recruitment of paracingulin and the RhoA activator 114RhoGEF into the junctions, and this complex increased the angiogenic potential of HDMEC [92]. Supporting this idea, the silencing of paracingulin gene (*Cgnl1*), greatly impaired tubule structure formation in 3D co-culture assays and diminished the number of vascular structures formed during vascular expansion in the developing retina, suggesting paracingulin as a defining factor in new vessel formation [93]. Furthermore, using baculovirus-infected insect cells, Vasileva et al. provided evidence for the interaction of paracingulin with MTs, which is important for the formation of both strong AJ and focal adhesions to ensure stabilization and further elongation of neovascular tubules [93,94]. Together, these results suggest that paracingulin might control endothelial cell proliferation and angiogenesis through the regulation of MT and RhoA activity.

## 6. Transmembrane TJ Proteins in the Control of Cell Proliferation

### 6.1. Claudins

Claudins are transmembrane proteins that contribute to paracellular transport by forming ion selective barriers and pores in a tissue-specific manner. As mentioned previously, a hallmark of EMT includes loss of barrier properties. Indeed, claudin dysregulation is commonly found in human carcinomas, often with decreased *CLDN* gene expression. Further, claudin overexpression can sometimes reverse the malignant phenotype. In lung squamous cell carcinoma, the transfection of claudins -5, -7, and -18 was able to suppress proliferation and inhibit G1/S transition associated with inhibition of AKT phosphorylation [95] (Figure 3).

However, some cancer studies reveal a correlation between claudin overexpression and tumor progression [96,97,98,99]. Examples include claudin-3 expression, which has been correlated with increased tubulogenesis and bromo-deoxy uridine (BrdU) incorporation in mouse inner medullary collecting duct cells (mIMCD-3) [100]. Similarly, overexpression of claudin-6, -7, or -9 enhanced invasiveness and proliferation of an adenocarcinoma cell line [96], and *CLDN4* and *18* were upregulated in pancreatic cancer tissues [101,102]. Claudin-18 isoform a2 is highly expressed in gastric, esophageal, pancreatic, lung, and ovarian cancers, and while it is considered as a putative marker, its exact role in tumor progression remains unknown [103]. Importantly, the high expression of claudins in neoplastic tissues does not coincide with border localization or barrier regulation. For example, claudin-4 is highly expressed in poorly differentiated pancreatic cancer cells and is enriched at basolateral membranes rather than the apical junctional complex [104]. Claudin-11 overexpression has been associated with proliferation and migration of oligodendrocytes in a mechanism dependent on its interaction with outer surface protein-associated protein 1 (OAP1) and β1-integrin [105] (Figure 4). Moreover, claudin-2 has been found localized at the nuclear fractions in highly proliferative lung adenocarcinoma cells [106].

The molecular consequences of claudin overexpression in cancer biology have not been clearly defined. In human liver cells, it has been suggested that the activation of the protein kinase complex c-Abl/PKCδ (PKC isoform delta) is critical for the acquisition of a malignant phenotype induced by claudin-1 overexpression (Figure 4) [99]. In this study, the overexpression of claudin-1 in normal liver hepatocytes led to an increased expression of metalloproteinsases (MMPs), promoting an invasive phenotype. Similar results were found in human melanoma cells where PKC activation by phorbol myristic acid (PMA) increased *CLDN*1 transcription and contributed to invasion [107]. Recent evidence also indicates that ephrin (Eph), through its EphB1 receptor, can control epithelial transformation through interaction with claudin-1 and -4. This interaction promotes EphB1 tyrosine phosphorylation, which in cancer cells mediates migration and invasion through the downstream exocytosis of MMPs [108]. Importantly, claudins can also be phosphorylated by Eph receptors that regulate cell-contact formation [108,109]. A role of claudin-2 in proliferation has been also suggested in studies with lung adenocarcinoma cells. In this work, it was proposed that claudin-2 could induce proliferation when it is phosphorylated at Ser208 and located at the nuclear fractions, interacting with ZO-1, ZONAB, and CycD1 [106] (Figure 3). Moreover, there was a positive correlation between claudin-4 expression, production of interleukin 8 (IL-8), and increased angiogenesis in mouse xenografts [110].

The *Cldn15* knockout mouse model suggests that alterations in barrier function might lead to homeostatic changes that activate other cellular processes including proliferation. *Cldn15* null mice have an enlarged upper small intestinal phenotype or mega-intestine due to the enhanced proliferation of the crypt cells [111]. In this knockout model, the expression and localization of claudins-1, -2, -3, -4, -7, -12, -18, -20, and -23 were not affected, but barrier properties were clearly lost. The authors concluded that the increased cellular proliferation is a consequence of altered barrier function that changes the intestinal environment [112]. This alteration in cell proliferation associated with an altered microenvironment may also be observed in metastasis studies of pancreatic carcinomas where the primary tissue has low expression of *CLDN3* while the metastatic cells that invade the liver express high levels of *CLDN3* and *4* but show low expression of *CLDN1* and *7* [113,114]. The altered TJ expression might influence the cancer microenvironment, promoting changes in cancer metastasis. Similar to *Cldn15*, *Cldn18* knockout mice show proliferation changes with lung enlargement, parenchymal expansion, and increased abundance and proliferation of known distal lung progenitors, the alveolar epithelial type II (AT2) cells, activation of Yes-associated protein (YAP), and increased organ size and tumor genesis in mice [115]. Importantly, claudin-18 and YAP were found to interact and co-localize at cell contacts in control samples (Figure 1B), and claudin-18 overexpression decreased YAP nuclear localization, suggesting that similar to ZO proteins, claudin-18 restricts cell proliferation by the retention of YAP at cell contacts.

Together, these data suggest that altered claudin expression, organization or function at the barrier may be observed as a hallmark of EMT and in a number of epithelial cancers. However, some cancers involve increased expression of specific claudins despite loss of barrier function, and overexpression of some claudins can alter the cancer cell phenotype. While loss of barrier function clearly alters the microenvironment of the cancer, the precise role of claudin in proliferation and cancer remains an area that requires additional investigation. Although claudins are promising molecular targets for diagnosis and therapy [102,116], a better understanding of the mechanisms regulated by claudins in the control of cell proliferation and metastasis is needed.

### 6.2. Junctional Adhesion Molecules (JAM)

JAM is the most extensively studied single-span TJ protein, and there are three genes described that encode to the proteins: JAM-A, -B, and -C. JAMs belong to the immunoglobulin superfamily because they contain at least one immunoglobulin domain at the extracellular N-terminus, while the cytoplasmic tail contains a PDZ binding sequence that interacts with the PDZ domain of ZO-1. 

JAM-A regulates epithelial proliferation via canonical Wnt signaling. In control mice, JAM-A shows a gradient expression along the intestinal crypt-luminal axis, which is increased in non-proliferating luminal epithelial cells [117]. JAM-A gene (*F11r*) deletion in mice results in increased intestinal epithelial cell proliferation and activation of AKT and its downstream target β-catenin, which is phosphorylated at Ser552 (Figure 3). This in turn, promotes β-catenin accumulation and its nuclear localization that is followed by an increase in T-cell factor (TCF)/LEF-induced transcriptional activity [118]. Moreover, a recent study revealed that JAM-A also regulates the cortical localization of dynein to control planar spindle orientation during mitosis via activation of Cdc42 and phosphatidyl inositol 3-kinase (PI3K) [119]. As expected, JAM-A suppression or expression of a dimerization-deficient isoform resulted in aberrant spindle orientation.

JAM-A dimerization promotes epithelial and endothelial migration. Expression of the JAM-A dimerization-defective mutant in 293T cells and the use of JAM-A dimer-disrupting antibodies reduce cell migration. Disruption of JAM-A dimerization also correlates with β1-integrin degradation, decreased GTPase Rap1 activation, and diminished numbers of focal concentrations of phosphorylated paxillin [120]. This process is mediated by the interaction of JAM-A with AF6 and PDZGEF2 [121,122]. Similar to these studies, isolated endothelial cells from mice deficient in *F11r* have enhanced spontaneous and random motility associated with increased numbers of actin-containing protrusions, reduced MT stability, and impaired focal adhesions, which can be reversed by JAM-A expression or by using glycogen synthase kinase 3-beta (GSK3β) inhibitors [123]. Moreover, mice deficient of JAM-C gene (*Jam3*) show increased *F11r* expression and enhanced retinal vascularization [124,125], supporting a role for JAM in angiogenesis.

Together, these data indicate that JAMs contribute to cell proliferation and migration. However, further studies are clearly needed in order to determine the exact role of JAMs in these processes.

### 6.3. MARVEL Family Proteins

**Figure 5:**
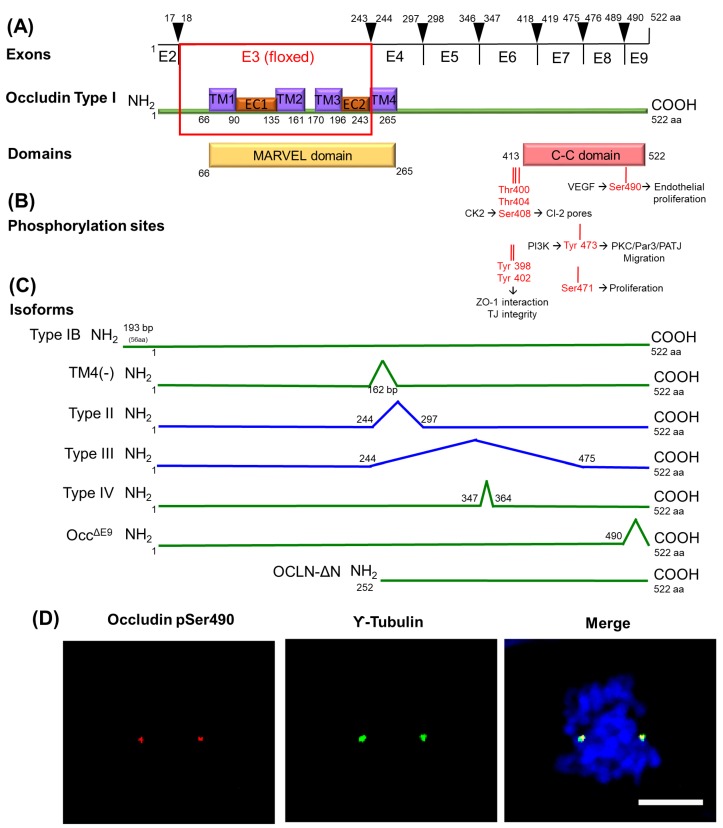
Occludin structure and isoforms. Occludin is a 522 amino acids protein encoded by 9 exons (**A**). Occludin full length (type I) possesses four transmembrane (TM) domains and two extracellular (EC) loops with the MARVEL domain as homology on the cytoplasmic side after each TM region. At the C-terminus (COOH) of occludin, a coiled-coiled (C-C) domain can be phosphorylated at multiple sites (**B**). Phosphorylation of occludin identified and known or implied functions. Occludin can mediate proliferation though the phosphorylation of two sites: Ser471, which regulates post contact proliferation in epithelial cells, and Ser490, which is promoted by VEGF-induced PKCβ activation and regulates both endothelial permeability and neovascularization. To date, several occludin isoforms have been described (**C**). The function of each isoform has not been fully elucidated, but most isoforms localize to the junctions except type II and III (blue lines). Interestingly, occludin deleted in exon 9 (Occ^∆E9^) restricts cell migration. (**D**) In bovine retinal endothelial cells, occludin stained with a pS490-specific antibody (red) shows co-localization of phospho-occludin with the centrosome marker γ-tubulin (green) in pro-metaphase. Hoechst dye (blue). Scale bar = 5 µm.

The MARVEL (for MAL and related proteins for vesicle trafficking and membrane link) family members include occludin (MarvelD1), tricellulin (MarvelD2), and MarvelD3 [126,127,128]. MARVEL proteins possess a putative protein-lipid-interacting motif containing four intramembrane helices creating the MARVEL domain (Figure 5A). Its function remains unknown, but MARVEL domains of non-TJ proteins, such as myelin and lymphocyte protein (MAL), have been associated with the generation and stabilization of functional membrane domains via the propensity for homo-oligomerization and the ability to attract apical membrane lipids [129]. Knockout studies of TJ MARVEL genes reveal a complex phenotype in a variety of tissues (reviewed in [130]). Many tissues are able to form morphological and functionally normal TJs, suggesting that they are not individually required for TJ formation although duplication of function has yet to be fully explored. Indeed, in vitro experiments demonstrate depletion of occludin gene (*Ocln*) resulting in the redistribution of tricellulin [131]. *Ocln* knockout mice develop hyperplasia of the gastric epithelium and testicular atrophy, while deletion of *MarvelD2* or *Ocln* in mice present degeneration of cochlear hair cells that leads to progressive hearing loss [132,133]. Moreover, Tricellulin is essential for barrier formation and the maintenance of the TJ structure in the inner ear as determined by gene deletion studies [126,134]. Additionally, MARVEL proteins have been found in membrane raft domains with a high cholesterol content, suggesting that they might participate in bending during the formation of endocytic caveolin transport vesicles similar to other proteins with MARVEL domains [135]. To our knowledge, there is no evidence for tricellulin in the control of cell proliferation, and we will focus on occludin and MarvelD3.

Occludin was the first transmembrane protein discovered at the TJ [136]. While the *Ocln* -null mouse forms intact TJs, the animals have phenotypic alterations including growth retardation, thinning of compact bone, testicular atrophy, male infertility, loss of cytoplasmic granules in salivary epithelial cells, females are not able to lactate, brain calcification [132], and hyper proliferation of mucous epithelial cells in the intestinal lining [137]. In vitro, the silencing of *OCLN* gene has a limited effect on barrier properties, with increases in permeability to divalent organic cations and also to small molecules under hydrostatic pressure [138,139]. In ARPE-19 cells, a human retinal pigmented epithelial cell line, loss of *OCLN* increases the DNA synthesis rate and cell proliferation [139]. Moreover, during neurogenesis, occludin loss has been found in neural tubes of chicken and mouse embryos at E9 [140].

Occludin function is regulated by phosphorylation. Specially, the C-terminal region has been studied extensively (Figure 5B). The 3 sites Thr400, Thr404, and Ser408 that lie just prior to the coiled-coil (C-C) domain regulate its interaction with ZO proteins and TJ integrity [141]. While occludin phosphorylation at Thr404 regulates its localization at the junctions [142], phosphorylation of Ser408 promotes ion flux through control of claudin-2 dimerization. The complex formed by occludin/ZO-1/claudin-2 is dissociated when occludin is phosphorylated by casein kinase 2 (CK2) at Ser408. As a result, claudin-2 interacts in trans with claudin-2 from neighboring cells, forms a cation pore, and increases ion flux [143]. Other phosphorylation sites nearby including Tyr398 and 402 residues may also alter ZO-1 binding [144]. Ser490 phosphorylation controls vascular endothelial growth factor (VEGF)-induced endothelial permeability with the expression of S490A point mutants preventing VEGF-induced permeability in cell culture. In endothelial cells, this results in increased vascular permeability [145] in a PKC isoform beta (PKCβ)-dependent manner [146].

Collectively, these studies demonstrate that occludin phosphorylation contributes to regulation of barrier properties by promoting its binding to ZO proteins and the recruitment of occludin and claudins into the junctions.

Occludin phosphorylation has also been associated with the regulation of cell proliferation and migration. The phosphorylation at Tyr473 promotes directional migration of epithelial cells by the activation of PI3K signaling and by promoting the organization of the aPKC, PAR3, and PATJ polarity complex [147], suggesting a role of occludin in cell migration. In addition, previous studies in our group have identified five phosphorylation sites on the C-C domain of occludin, including Ser471 and Ser490, mentioned above, on the two turns of the C-C domain, which have been associated with the control of cell proliferation. Expression of the S471A mutant as a stable cell line has no effect on sub-confluent proliferation but inhibits proliferation and cell packing after cell contact in MDCK cells as determined by cell number and DNA synthesis, leading to enlarged cells. Inhibition of proliferation by expressing S471A point mutant occludin, inhibition of cell proliferation in cell contacted immature monolayers or inhibition of the Ser471 kinase G-protein coupled receptor kinase (GRK), all profoundly inhibit TJ formation and epithelial monolayer maturation [148]. The Hippo signaling pathway is an important determinant of cell and organ sizes, and nuclear exclusion of the co-activator YAP accompanies proliferative quiescence. The Hippo pathway elements YAP and TEAD (TEA-dependent) have been found to co-localize with occludin in pancreatic cancer cells and regulate cell proliferation [149].

The second phosphorylation site of occludin that regulates cell proliferation was identified at Ser490. This phosphorylation can be induced by VEGF and promotes occludin ubiquitination and its intracellular trafficking. In endothelial cells, this results in increased vascular permeability [145] in a PKCβ-dependent manner [146]. Moreover, studies on the phosphorylation of this site have revealed a novel function of occludin as a regulator of centrosome separation and mitosis initiation. In MDCK cells, the expression of occludin mutated at Ser490 to Ala slows cell proliferation and hindered mitotic entry due to delayed centrosome separation. Stable expression of aspartic acid phosphomimetic (S490D) in MDCK cells results in centrosomal localization of occludin and cell proliferation. However, expression of the nonphosphorylatable alanine mutation (S490A) of occludin impedes centrosome separation, delays mitotic entry, and reduces proliferation. Collectively, these studies demonstrate a novel location and function for occludin in centrosome separation and mitosis [150]. Similar results were found in endothelial cell culture and in retinal tissue, where the induction of endothelial-specific expression of the occludin S490A mutant through viral delivery completely inhibited neovascularization [151]. In this study, primary bovine retinal endothelial cells in collagen matrices responded to VEGF with increased Ser490 phosphorylation coincident with tube formation. Transfection of occludin with the S490A point mutant inhibited tube formation, proliferation, and migration compared to wild-type occludin. Western blotting revealed increased occludin phosphorylation associated with angiogenesis, and whole-mount immunofluorescent staining of the retinas revealed centrosomal occludin organization in proliferating vessels. Further, mice with doxycycline-inducible *VEGF* expression from photoreceptors were used to study occludin control of angiogenesis in vivo. Expression of the occludin S490A mutant by sub-retinal injection of adeno-associated virus significantly reduced retinal vessel growth in vivo. Importantly, in these studies, occludin was located at the centrosomes (Figure 5D), and increased occludin phosphorylation was found in dividing endothelial cells, mouse retinas with neovascularization, and human surgical samples of retinal neovessels, suggesting a novel role for occludin in regulation of endothelial proliferation and neovascularization in a phosphorylation-dependent manner

Similar to claudins, previous publications have demonstrated that oncogenic transformation of a variety of cell types is associated with altered occludin expression [152]. *OCLN* gene is downregulated in premalignant foci in kidneys from patients with germ line tumor suppressor von Hippel–Lindau (*VHL*) gene mutations [153]. At a molecular level, it has been suggested that *OCLN* is transcriptionally repressed following constitutive Raf-1 expression [154] this is mediated through a direct interaction between activated Slug and the E-box in the *Ocln* promoter [155]. Similarly, in murine melanoma cells, *Ocln* is epigenetically silenced through promoter hyper-methylation, and its forced expression also reduces tumor migration. This is supported by other studies suggesting that occludin possess anti-tumorigenic properties [156,157]. The expression of exogenous occludin suppresses tumor growth in nude mice of Raf1-transformed rat salivary gland epithelial cells [158]. Similarly, stable occludin expression in melanoma and breast cancer cells followed by injection into the craniolateral thorax and mammary fat pad, respectively, reduced the size of lung metastases [157]. In a cell culture model of uveal melanoma, blood vessel epicardial substance (BVES) protein overexpression led to an increase in ZO-1 and occludin, which correlated with decreased cell proliferation [159]. Further, occludin induces premature senescence in breast cancer cells, which can be blocked by chemical inhibition of the mitogen-activated protein kinase (MEK) pathway [160]. Conversely, occludin has been also implicated in EMT, where occludin targets the TGFβ receptor to the junctional complex and promotes efficient epithelial transformation [161] (Figure 2), a mechanism that correlates with the simultaneous repression of the genes encoding E-cadherin, claudins, and occludin [39]. Finally, *OCLN* depletion in MDCK cultures demonstrated impaired mitotic spindle orientation due to a reduced interaction with ZO-1, suggesting an alteration of the cues necessary for polarization in cell division [162].

Other studies support a role of occludin in proliferation and interaction with centrosomal proteins. *OCLN* mutations in human patients can lead to microcephaly and band-like calcifications with polymicrogyria characterized by loss of cortical convolutions, shallow or absent sulci, and multiple small gyri giving the cortex surface a roughened irregular appearance [163,164,165,166,167,168]. To date, thirteen pathogenic mutations in *OCLN* gene have been identified in thirteen families, and seven mutations are situated in exon 3 [165,166,167,168]. Primary microcephaly (MCPH, for microcephaly primary hereditary) is a disorder of brain development that results in a head circumference more than three standard deviations below the mean for age and gender. Notably, many of the causative genes for MCPH encode centrosomal proteins involved in centriole biogenesis [169]. 

Recently, a new isoform of occludin was discovered by Bendriem et al. with a specific function in the proliferation of human embryonic stem cells (hESCs) and in neural progenitors that alter cortex size in the developing mouse brain (Bendriem et al., accepted for publication in ELife). The original *Ocln* knockout mouse line was generated by excising exon 3 and was believed to be a null model. While mouse full-length occludin (OCLN-FL) is no longer expressed, a truncated form that lacks its N-terminus and three of its four transmembrane domains (OCLN-ΔN) is still expressed. This is a 32-34 kDa protein that results from a shorter ΔN transcript lacking exons 2 and 3 (Figure 5C). Both OCLN-FL and OCLN-ΔN isoforms localize to the centrosomes; however, in the homozygous mutant mouse line *Ocln*^ΔN*/*^^ΔN^, OCLN-ΔN localizes to interphase and mitotic centrosomes in the embryonic mouse cortex but not at the plasma membrane, suggesting the C-terminal domain of occludin is important for this centrosomal localization. Consistent with *OCLN* mutation in patients with microcephaly, depletion of *Ocln* in mice led to microcephaly. An increased mitotic index was found in *Ocln*^ΔN*/*^^ΔN^ mutant mice along with a higher percentage of cells of *Ocln*^ΔN*/*^^ΔN^ E12.5 cortices in prometaphase and metaphase compared to the wild-type. Moreover, prolonged mitosis led to a higher percentage of activated (cleaved) caspase 3 (CC3)-positive apoptotic cells in mutant embryos compared to controls prior to E14.5. 

Occludin centrosomal localization was also confirmed in vitro in two hESCs lines that closely resemble the *Ocln*^ΔN*/*^^ΔN^ mouse mutant. Mutant hESC-derived organoids displayed pronounced proliferation defects, premature differentiation, and apoptosis. Cells numbers with a reduced ratio of the basal neural progenitor marker HOPX (homeodomain-only protein homeobox) compared to the early neuronal marker NeuroD1 may be responsible for the reduced size of human organoids. Importantly, centrosomal occludin co-localized and immune-precipitated with NuMA (for Nuclear Mitotic Apparatus protein) and the small GTPase RAN (RAs-related Nuclear protein), two important proteins in mitotic spindle assembly and stabilization, and mutant hESCs exhibited impaired mitotic spindles and abnormal morphology at the spindle poles. These studies demonstrated an important role for occludin in neurogenesis through its centrosomal interactions and promotion of proper functioning neural progenitor mitotic spindles.

Along with occludin localization at the centrosomes, studies have also shown that intracellular occludin-containing vesicles move along MTs and contribute to the regulation of cell proliferation [170]. MTs interacting with plasma membranes participate in the preservation of epithelial TJ structure and function. This is regulated by the binding of MT plus end–tracking proteins at the scaffold in the AJs or may be achieved through MT minus end binding of nezha/calmodulin-regulated spectrin-associated protein (CAMSAP) and ninein to the AJs [171,172,173,174,175]. The junctional localization of several MT organizing center proteins indicates the crucial role of junctions as sites that orchestrate MT organization in polarized cells [94]. Glotfelty et al. [170] reported that intracellular occludin-containing vesicles move along MTs and that the rate of movement depends on intact MT networks. This suggests that dynein, a key regulator of MT-vesicle trafficking, may regulate this process in the minus-end direction. Consistent with this hypothesis, the siRNA knockdown of dynein/dynactin induced occludin accumulation in the cytosol, whereas plus-end motor kinesin knockdown did not. This model of MT-dependent TJ trafficking was further supported by the results from studies on dynein and Rab11 [176]. Rab11 utilizes MTs for trafficking and has been shown to participate in occludin trafficking by the regulation of the Rab11 FIPs (Rab11 family interacting protein) [176]. 

Occludin-containing vesicles may traffic bi-directionally on MTs to regulate cell proliferation. Previous studies suggest that MTs traffic likely contribute to TJ assembly by functioning as tracks for the delivery of TJ proteins through MT-associated vesicles. Particularly occludin-containing granules have been found near the tip of oolemma ingression in dividing *Xenopus* oocytes [177]. Rab13 or junction rab (JRab) is a key mediator of the endocytic recycling of occludin [178] through its binding partners Rab13-binding protein and MICAL-like protein 2 (MICAL-L2) [179,180]. VAP-33 (VAMP associated protein of 33 kDa) is implicated in vesicle docking/fusion and binds to occludin, and its overexpression promotes occludin movement along the lateral edge of the plasma membrane [181]. Identification and characterization of a homolog of VAP-33 in *Drosophila* (DVAP-33A) revealed that DVAP-33A regulates the division of boutons at the synaptic terminals by stabilizing and directing the MT cytoskeleton during budding [182]. Thus, TJ assembly mediated by occludin-MT interaction may play an important role in both cell proliferation and junction organization.

Together, these studies indicate that occludin both regulates barrier function by controlling TJ protein internalization and localizes at centrosomes, contributing to the regulation of cell proliferation.

MarvelD3 is identified as the third member of proteins with a MARVEL domain [128]. MarvelD3 is expressed as two isoforms that show a broad tissue distribution. The two isoforms represent splice variants and share the predicted N-terminal cytoplasmic domain of 198 amino acids, but differ in their C-terminal halves that contain the transmembrane domains. MarvelD3 does not have the long cytoplasmic C-terminus ending in a C-C domain found on occludin and tricellulin. RNA interference experiments in steady-state Caco-2 monolayers indicate that MarvelD3 is required to maintain epithelial integrity under osmotic stress, but this is not essential for TJ formation [183]. However, Raleigh et al. reported that knockdown of *MARVELD3* delays TJs assembly in Caco-2 cells [127]. Further, depletion of *MARVELD3* by siRNAs in the human pancreatic cancer cell line (HPAC) resulted in downregulation of barrier function as shown by decreased electrical resistance and increased permeability to fluorescent dextran tracers, whereas knockdown did not affect the fence function of TJs maintaining apical and basolateral membrane protein restriction [184]. Interestingly, in a genome-wide association study, an intergenic single nucleotide in *MARVELD3* correlated with resistance to severe malaria [185] while its specific role has not yet been discovered. 

Recent studies suggest a role of MarvelD3 in EMT, cell proliferation, and migration. In tumor cells, MarvelD3 was downregulated during EMT in human pancreatic cancer cells [184]. Similar effects were also observed when MCF-7 breast cancer cells that are known to express MarvelD3 were compared to MiaPaca-2 pancreatic tumor cells, which do not express detectable MarvelD3. In this study, transfection of MarvelD3 in MiaPaca-2 cells reduced cell migration and proliferation. When they were injected in a xenograft model, MarvelD3 overexpressing MiaPaca-2 cells revealed reduced tumor volume as compared to cells with low MarvelD3 expression. Moreover, siRNA-mediated depletion of *MARVELD3* induced Caco-2 cell migration and proliferation, and the effects could be rescued by expressing mouse MarvelD3. These data indicate that MarvelD3 regulates cell proliferation and cell migration of differentiating and dedifferentiated epithelial model cell lines. The authors suggest that MarvelD3 functions as a signaling transmembrane component of TJs. 

MarvelD3 activates MEKK1/JNK (mitogen-activated protein kinase kinase 1/c-Jun NH_2_-terminal kinase) signaling through its N-terminal cytoplasmic domain to control cell proliferation. [183]. The interaction of the N-terminal domain of MarvelD3 with upstream component MEKK1 that regulates JNK activation was first demonstrated in glutathione S-transferase (GST) pull-down assays in Caco-2 cells. Further studies in MDCK cell lines expressing MarvelD3 fusion proteins carrying the biotin ligase at both the C-terminus and N-terminus supported this conclusion and corroborated the specific interaction of MEKK1 with the N-terminal domain of MarvelD3. Moreover, in MarvelD3 overexpressing MiaPaca-2 cells, MEKK1 was partially recruited to cell–cell contacts when MarvelD3 was expressed in MiaPaca-2 cells but not in control cells, indicating that re-expression of MarvelD3 is sufficient to stimulate membrane recruitment of MEKK1. 

MarvelD3 modulates cell proliferation and migration in early embryogenesis. A recent study with *Xenopus* indicated that MarvelD3 modulates cell proliferation in early eye development and regulates cell survival during eye morphogenesis [186]. In these studies, the JNK pathway was required for proper eye morphogenesis by acting as an inhibitor of the expression of eye-field transcription factors (EFTFs). EFTFs specify a single eye-field in the most anterior region of the neural plate. In this region, inhibition of cell-cycle activators occurs to favor EFTF expression, while duration of the expression of the transcription factors is established by cell-cycle-independent factors. The single eye-field is then divided into two eye primordia under the influence of Sonic hedgehog signaling. Moreover, MarvelD3 is part of a regulatory feedback loop that coordinates JNK activity with neural crest formation. *MarvelD3* depletion enhances JNK signaling, which leads to disruption of neural crest derivative differentiation and neural crest precursor formation, as well as displacement of the neural plate border. Consistent with this model, inhibition of JNK signaling is sufficient to rescue the phenotype induced by *marvelD3* depletion, while constitutively active JNK disrupts neural crest development, supporting the importance of controlled regulation of JNK activity [187]. Together, these data present a novel role of MarvelD3 as an essential regulator of early vertebrate development and neural crest induction that relies on interplay between gene expression, cell proliferation, and cell migration.

## 7. Conclusions

While numerous studies clearly demonstrate the role of TJ proteins in barrier regulation, more recent research suggests a number of tight junction proteins also contribute to the control of cell proliferation and growth. The junction proteins may contribute to proliferation in a number of mechanisms. In some cases, control of barrier properties alters the microenvironment contributing to growth control. Further, TJ proteins may localize transcription factors and cell cycle control proteins at the plasma membrane, restricting nuclear access or inhibiting function. In the presence of growth factors, cell polarization is disrupted and the TJ proteins release these factors, allowing progression through the cell cycle. In addition, a number of TJ proteins demonstrate non-typical cellular localization associated with cell proliferation, revealing distinct and unique functions from their role in cell-cell contact. TJ proteins have been found localized in the nucleus at G1/S cell cycle transition, associated with integrins at the basal membrane during migration, or in mitosis interacting with microtubules or with centrosome proteins. Together, these studies reveal the coordination of epithelial and endothelial barrier formation with cell proliferation, an essential component of cellular growth and differentiation.

## Abbreviations

α-SMA AF6 AJ AT2 BBB BMP BRB BrdU BVES CAMSAP C-C CC3 CCM Cdk4 CK2 Cl CNS CycD1 DLG EC EFTFs EGF EGFR2 or ERBB2 EMT EndMT Eph EphB1 FGF FSP1 FIP Fzd4 GEF-H1 Gpr124GRK GSK3β GST HDMEC hESCs HGF HOPX HPAC IL-8 JACOP JAM JNK JRAB LEF LGL Lrp5/6 MAGUK MAL MARVEL MCPH MDCK MEK MEKK MET MICAL-L2 mIMCD-3 MMP MT NuMA OAP1 Occ^∆E9^ OCLN-FL OCLN-ΔN PALS1 PAR PATJ *PCNA* PI3K PKC PLVAP PMA RAN Reck Sip-1 SJ Sox TCF TEAD TGFβ TGFβR Tiam1 TJ TM Tspan12 Ubq VAMP VAP-33 VE-cadherin VEGF VHL vWF YAP ZEB-1 ZO ZONAB	alpha smooth muscle actin afadin adherens junction alveolar epithelial type II cells blood-brain barrier bone marrow morphogenetic protein blood-retinal barrier bromo-deoxy uridine blood vessel epicardial substance protein calmodulin-regulated spectrin-associated protein coiled-coil domain cleaved caspase 3 cerebral cavernous malformation cyclin-dependent kinase 4 casein kinase 2 claudin central nervous system cyclin-D1 disc large extracellular eye-field transcription factors epidermal growth factor epidermal growth factor receptor epithelial-mesenchymal transition endothelial-mesenchymal transition ephrin ephrin B1 receptor fibroblast growth factor fibroblast-specific protein 1 family interacting protein frizzled-4 guanine nucleotide exchange factor H1 G-protein-coupled receptor 124 G-protein coupled receptor kinase glycogen synthase kinase 3 beta glutathione S-transferase human dermal microvascular endothelial cells human embryonic stem cells hepatocyte growth factor homeodomain-only protein homeobox human pancreatic cancer cell line interleukin 8 junction-associated-coiled-coil protein junctional adhesion molecule c-Jun NH_2_-terminal kinase junction Rab lymphoid enhancer-binding factor lethal giant larvae low density lipoprotein receptor-related protein 5/6 membrane-associated guanylate kinase homolog myelin and lymphocyte protein MAL and related proteins for vesicle trafficking and membrane link microcephaly primary hereditary Madin–Darby canine kidney mitogen-activated protein kinase mitogen-activated protein kinase kinase mesenchymal-epithelial transition MICAL-like protein 2 mouse inner medullary collecting duct cells matrix metalloproteinase microtubules nuclear mitotic apparatus protein outer surface protein -associated protein 1 occludin deleted in exon 9 occludin-full length occludin N-terminal deleted protein associated with lin seven 1 partitioning defective protein associated with tight junctions proliferating cell nuclear antigen phosphatidyl inositol 3-kinase protein kinase C plasmalemma vesicle-associated protein phorbol myristic acid Ras-related nuclear protein reversion-inducing cysteine-rich protein SMAD interacting protein 1 septate junction sex determining region Y-related high mobility group box factors T-cell factor TEA-dependent transforming growth factor-beta transforming growth factor-beta receptor T-cell lymphoma invasion and metastasis 1 tight junctions transmembrane tetraspanin-12 ubiquitination vesicle-associated membrane proteins VAMP associated protein of 33 kDa vascular endothelial cadherin vascular endothelial growth factor von Hippel–Lindau von Willebrand factor c-yes associated protein zinc finger E-box-binding homeobox 1 zona occludens ZO-1-associated nucleic acid binding protein
